# Cloning and Expression of Ecdysone Receptor and Retinoid X Receptor from *Procambarus clarkii*: Induction by Eyestalk Ablation

**DOI:** 10.3390/ijms17101739

**Published:** 2016-10-18

**Authors:** Tian-Hao Dai, Ali Sserwadda, Kun Song, Ya-Nan Zang, Huai-Shun Shen

**Affiliations:** 1 Wuxi Fisheries College, Nanjing Agricultural University, Nanjing 210095, China; qwedsa704@aliyun.com (T.-H.D.); alisserwadda61@gmail.com (A.S.); qwedsa705@aliyun.com (K.S.); qwedsa706@aliyun.com (Y.-N.Z.); 2 Key Laboratory of Freshwater Fisheries and Germplasm Resources Utilization, Ministry of Agriculture, Freshwater Fisheries Research Center, Chinese Academy of Fishery Sciences, Wuxi 214081, China

**Keywords:** ecdysone receptor, retinoid X receptor, *Procambarus clarkii*, molting, eyestalk ablation

## Abstract

Ecdysone receptor and retinoid X receptor are key regulators in molting. Here, full length ecdysone receptor (*PcEcR*) and retinoid X receptor (*PcRXR*) cDNAs from *Procambarus clarkii* were cloned. Full length cDNA of *PcEcR* has 2500 bp, encoding 576 amino acid proteins, and full length cDNA of *PcRXR* has 2593 bp, in which a 15 bp and a 204 bp insert/deletion splice variant regions in DNA binding domain and hinge domain were identified. The two splice variant regions in *PcRXR* result four isoforms: *PcRXR1*-4, encoding 525, 520, 457 and 452 amino acids respectively. *PcEcR* was highly expressed in the hepatopancreas and eyestalk and *PcRXR* was highly expressed in the eyestalk among eight examined tissues. Both *PcEcR* and *PcRXR* had induced expression after eyestalk ablation (ESA) in the three examined tissues. In muscle, *PcEcR* and *PcRXR* were upregulated after ESA, *PcEcR* reached the highest level on day 3 after ESA and increased 33.5-fold relative to day 0, and *PcRXR* reached highest the level on day 1 after ESA and increased 2.7-fold relative to day 0. In the hepatopancreas, *PcEcR* and *PcRXR* d*EcR*eased continuously after ESA, and the expression levels of *PcEcR* and *PcRXR* were only 0.7% and 1.7% on day 7 after ESA relative to day 0, respectively. In the ovaries, *PcEcR* was upregulated after ESA, reached the highest level on day 3 after ESA, increased 3.0-fold relative to day 0, and the expression level of *PcRXR* changed insignificantly after ESA (*p* > 0.05). The different responses of *PcEcR* and *PcRXR* after ESA indicates that different tissues play different roles (and coordinates their functions) in molting.

## 1. Introduction

The red swamp crayfish *Procambarus clarkii* is a freshwater crayfish species, native to the Southeastern region of the United States, and has been introduced to countries in Asia, Africa, Europe and other regions of the world. The species is said to have invaded China at the beginning of the 20th century [1]. Since the 1990s, the crayfish has been farmed widely and it has become an important aquaculture crustacean in the southeastern part of China, especially in Jiangsu Province [2].

Like all arthropods, crayfish have a thin, but tough exoskeleton which is shed regularly during development, a process most commonly referred to as molting. Molting, as the most striking feature in anthropods, is indispensable for many biological processes, including growth, reproduction and metamorphosis. During the molting stage, the red swamp crayfish are prone to attacks from other crayfish which may result into death, given the fact that their exoskeletons are weak. For this reason therefore, the red swamp crayfish is not a good subject for high-density farming.

Ecdysteroid, as a lipophilic small molecule, performs its function in the nucleus. It binds to a nuclear receptor complex, which is constituted of two nuclear receptors: ecdysone receptor and retinoid X receptor (*RXR*), or ultraspiracle (*USP*), the homologue of *RXR* in insects [3,4]. After binding ecdysteroid, the *EcR*-*RXR* complex is activated. It regulates transcription of target genes, such as E75 and chitinase [5,6]. Moreover, the *EcR* can regulate the transcription of its own gene, as well as *EcR* and *RXR* molt-responsive genes [7,8].

Both *EcR* and *RXR* have all the conserved nuclear receptor structures, including the A/B, C, D, and E/F domains [9]. Among these conserved domains, the C domain is the most conserved. It is a DNA-binding domain, which binds ecdysone responsive elements in the promoters of molting-responsive genes. The moderately conserved domain is the E domain, which is a ligand-binding domain and more complex than the other domains. Besides its major role of ligand binding, it also mediates heterodimerization and regulates ligand-dependent transcriptional activation (AF-2) [10]. The less-conserved D domain is the hinge domain and links the DNA binding domain and ligand binding domain. The N-terminal A/B domain and the C-terminal F domain are always highly variable [10].

To date, *EcRs* and *RXRs* have been reported from many crustaceans, including the *EcR* and *RXR* from the fiddler crab *Uca pugilator* [11], the *EcR* from the land crab *Gecarcinus lateralis* [12], the *EcR* and *RXR* from the kuruma prawn *Marsupenaeus japonicus* [13], the *EcR* and *RXR* from the water flea *Daphina magna* [14], the *EcR* from the intertidal copepod *Tigriopus japonicas* [15], the *EcR* and *RXR* from the brown shrimp *Crangon crangon* [16], the *EcR* from the mysid shrimp *Americamysis bahia* [17], the *EcR* and *RXR* from the American lobster *Homarus americanus* [18], the *EcR* from the harpacticoid copepod *Amphiascus tenuiremis* [19], the *EcR* from the blue crab *Callinectes sapidus* [20], the *EcR* and *RXR* from the opossum shrimp *Neomysis integer* [21] and the *EcR* and *RXR* from the freshwater prawn *Macrobrachium nipponense* [22,23] and the *EcR* from the Chinese mitten crab *Eriocheir sinensis* [24].

Isoforms of *EcRs* and *RXRs* are always found in crustaceans. The variant regions among these isoforms occur frequently in the A/B domain, the hinge domain and the ligand binding domain. These variations affect transcriptional activation, dimerization, and presumably ligand binding. For example, four isoforms of *EcR* from the freshwater prawn *Macrobrachium nipponense*, which differ in the hinge and ligand binding domain, exhibit sex-specific dimorphic expression patterns [22].

To improve the basic knowledge about molting in *P. clarkii*, here we cloned the full length cDNA of *EcR* and *RXR* gene from the red swamp crayfish *P. clarkii*. We also described the expression of *PcEcR* and *PcRXR* in different tissues and in response to eyestalk ablation.

## 2. Results

### 2.1. Cloning of Full Length cDNAs of PcEcR and PcRXR

**Figure 1:**
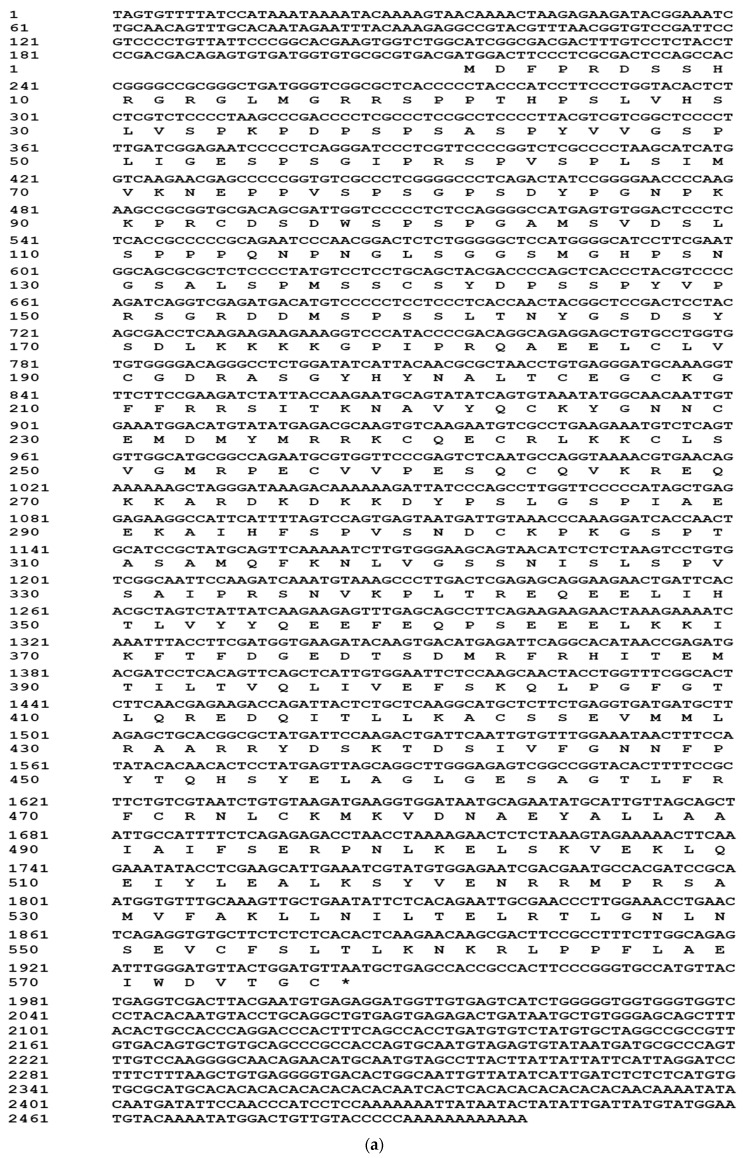

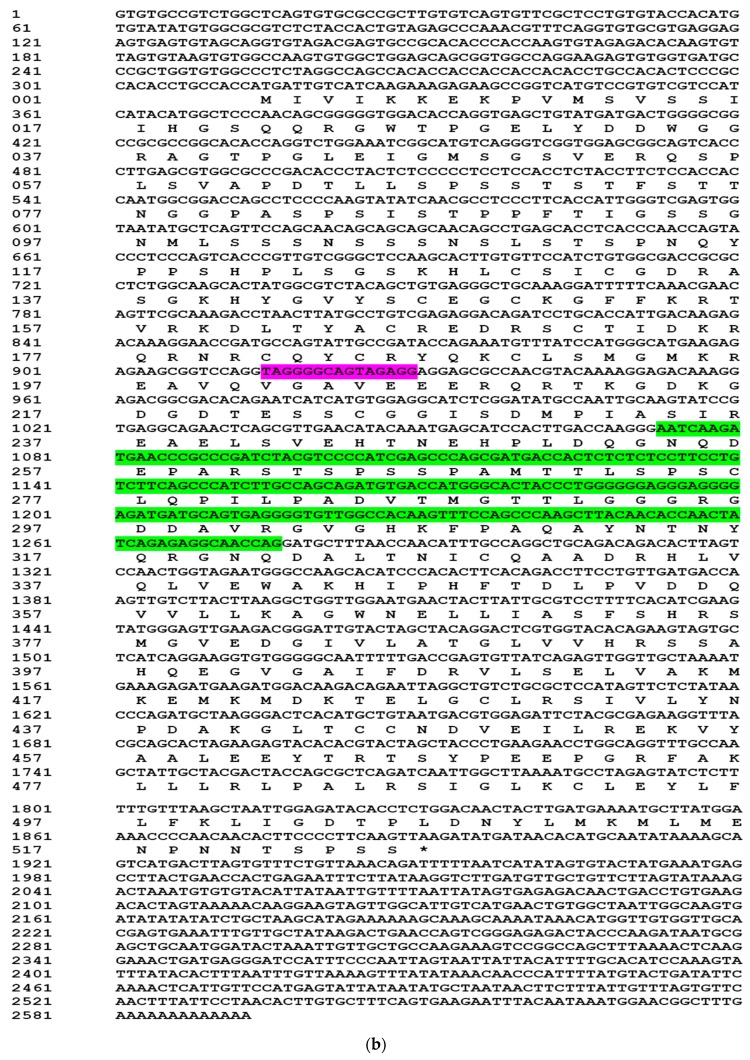
(**a**) Full-length cDNA of *PcEcR* and its encoded amino acid sequence; (**b**) Full-length cDNA of *PcRXR* and its encoded amino acid sequence. The two splice variant sequences: one with the red background is a 15 bp insertion/deletion alternatively spliced intron that only exists in *PcRXR1* and *PcRXR3*; the second with green background is a 204 bp insertion/deletion alternatively spliced intron that only exists in *PcRXR1* and *PcRXR2*.

Using 5′ and 3′ RACE, we isolated full length cDNA sequences of *PcEcR* (Figure 1a) and *PcRXR* (Figure 1b). The 2500 bp full-length cDNA of *PcEcR* (KX673814) consisted of a 213 bp 5′ un-translated region (UTR), a 556 bp 3′ UTR with a poly(A) tail and a 1731 bp open reading frame (ORF), which encodes a deduced 576 amino acid proteins. Four variant forms of *PcRXR* were identified, designated *PcRXR1*, 2, 3, and 4 (KX673813, KX673815, KX673816, KX673817). It consists of two insert/deletion regions, one is 15 bp and another is 204 bp among these *PcRXR* variants. 15 bp insert/deletion region is located in DNA binding domain and is present in *PcRXR1* and *PcRXR3*, and the 204 bp insert/deletion region is located in hinge domain and is present in *PcRXR1* and *PcRXR2*. The longest cDNA of *PcRXRs*, *PcRXR1*, is 2593 bp, consisted of a 313 bp 5′ un-translated region (UTR), a 702 bp 3′ UTR with a poly(A) tail and a 1578 bp open reading frame (ORF), which encodes a deduced 525 amino acid proteins.

### 2.2. Sequence Alignments and Phylogenic Trees of PcEcR and PcRXR

**Figure 2:**
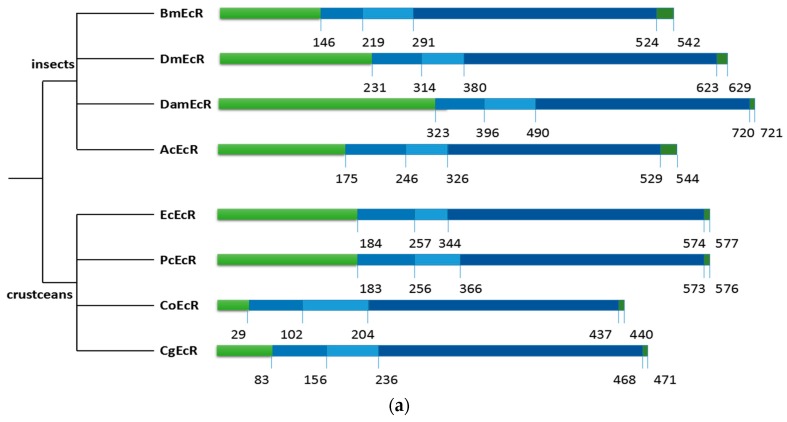

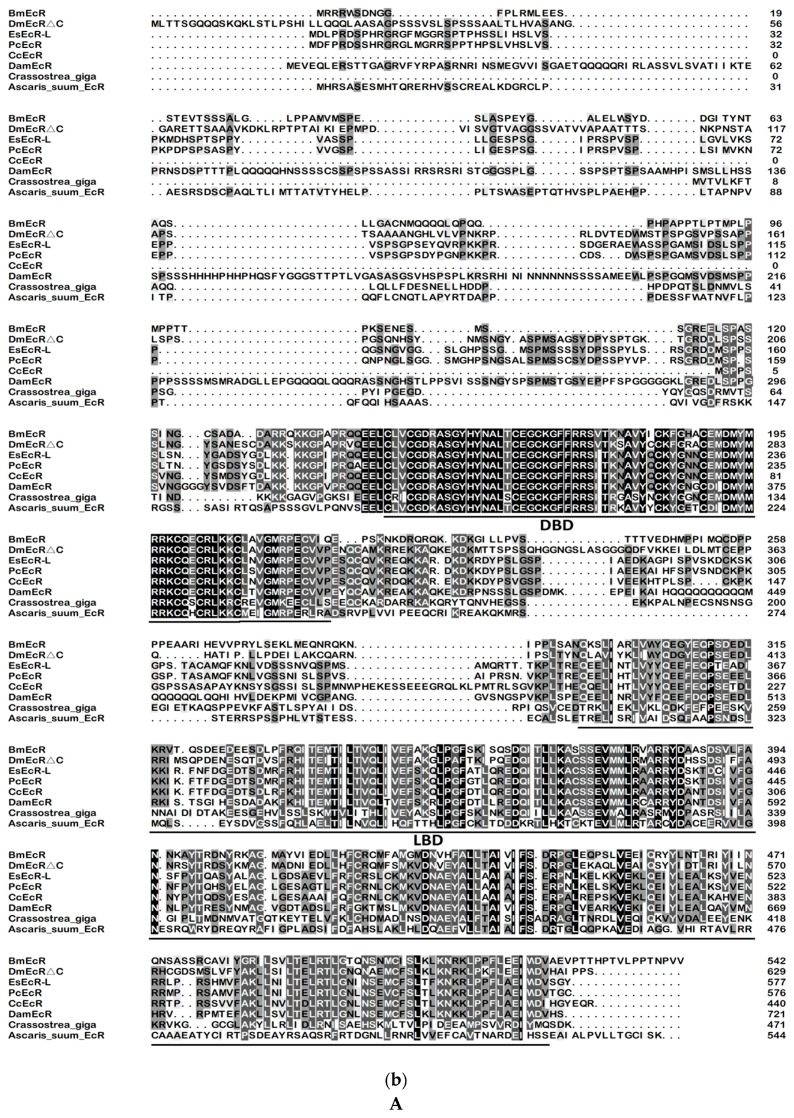

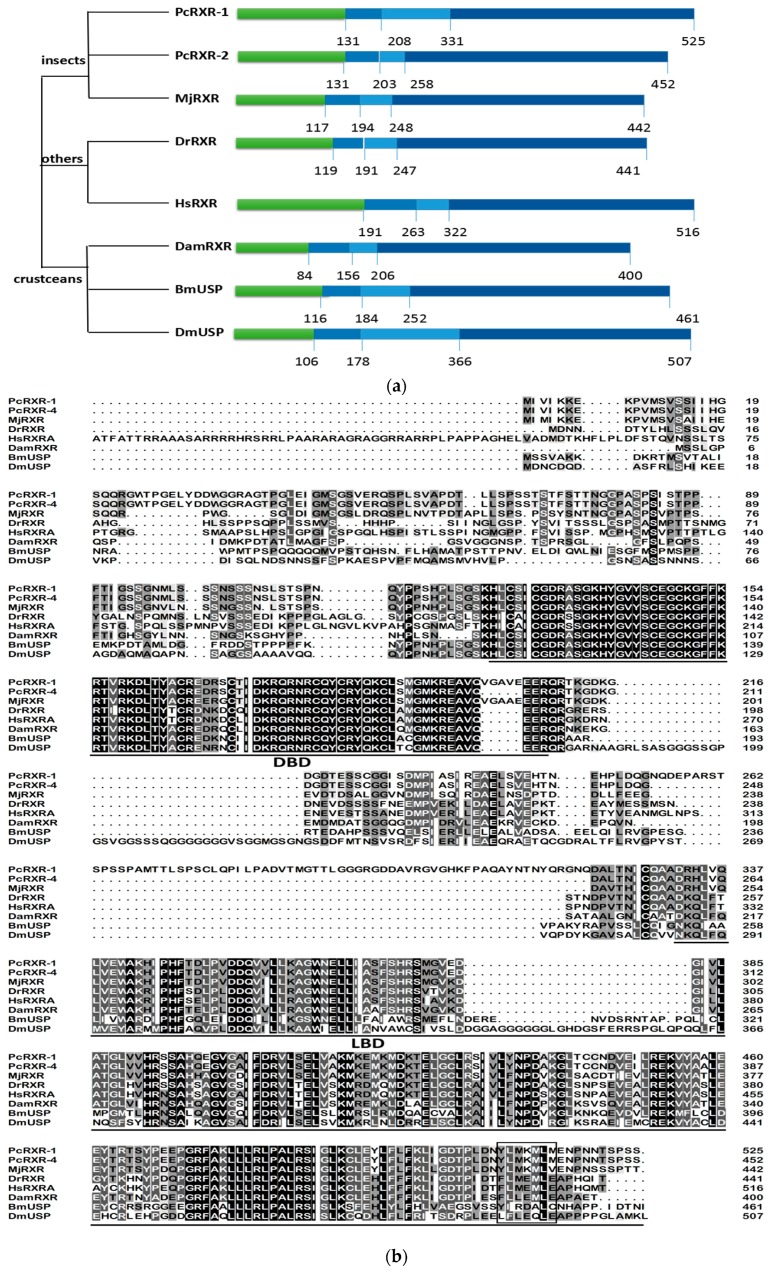

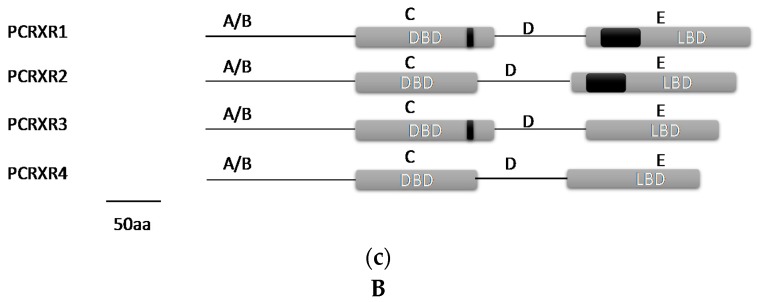
(**A**) The former figure (**a**) is a simple schematic representation of both transcripts highlighting each domain region. The latter figure (**b**) is a comparison between deduced amino acid sequences of *PcEcR* with other seven *EcRs*. Amino acid residues that are identical or similar between all sequences are highlighted. The conserved DBD (DNA-binding domain) and LBD (ligand-binding domain) domains are underlined. Sequence names and accession numbers are supplied in the Methods section; (**B**) The first figure (**a**) is a simple schematic representation of both transcripts highlighting each domain region. The second figure (**b**) is a comparison between deduced amino acid sequences of *PcRXR1* and *PcRXR4* with other six *RXRs*. Amino acid residues that are identical or similar between all sequences are highlighted. The conserved DBD and LBD domains are underlined. Sequence names and accession numbers are supplied in the Methods section. The third figure (**c**) is a simple alignment of each domain region with *PcRXR* 1,2,3,4. PcRXR has all the functional domains characteristic of nuclear receptors (A/B, C, D, E domains). The gray represents the conserved DBD and LBD domains and the black represents these variants in domain.

The alignment revealed that *PcEcR* and *PcRXR* has all the functional domains characteristic of nuclear receptors (A/B, C, D, E and F domains) (Figure 2). The C domain, which is the DNA-binding domain, is the most conserved in both *EcRs* and *RXRs*. DBD of *PcEcR* exhibits a high degree of identity (>86.3%) with the other *EcR* proteins, while DBD of *PcRXR* exhibits a high degree of identity (>82.6%) with the other *RXR* proteins. The E domain which is a ligand-binding domain, exhibit moderate conserved in both *EcRs* and *RXRs*. LBD of *PcEcR-1* shares a general degree of identity (>36.1%) with the compared *EcR* proteins, while LBD of *PcRXR-1* shares a general degree identity (>38.2%) with the compared *RXR* proteins. The most variable domains are the N-terminal A/B domain and the C-terminal F domain (Figure 2).

**Figure 3:**
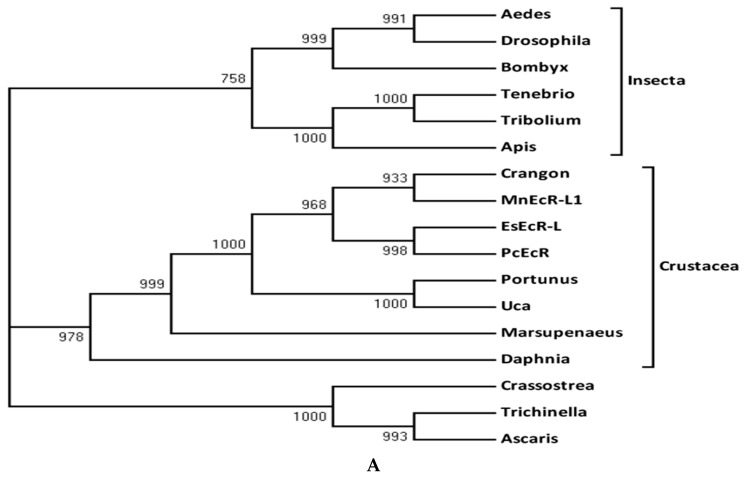

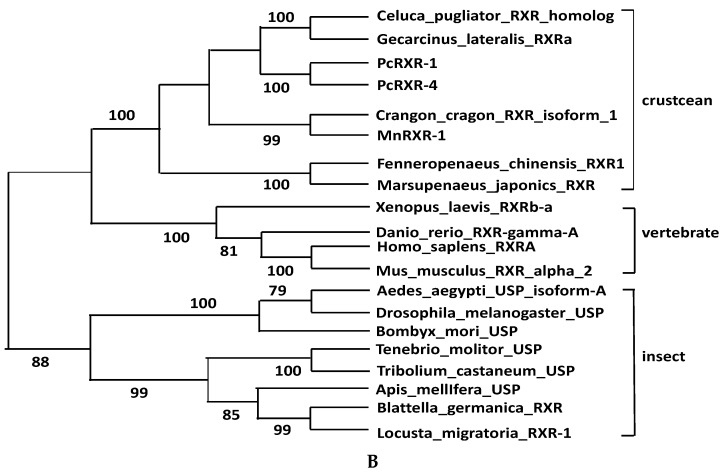
(**A**) Phylogenetic tree of *EcRs*. The tree was constructed using the neighbor-joining method. Numbers represent bootstrap values (%). Sequence names and accession numbers are supplied in the Methods section; (**B**) Phylogenetic tree of *RXRs*. The tree was constructed by use of the neighbor-joining method. Numbers represent bootstrap values (%). Sequence names and accession numbers are supplied in the Methods section.

In the phylogenetic tree of *EcRs*, the crustacean group is clustered in one clade and the insect group in another (Figure 3a). In the phylogenetic tree of *RXRs*, *PcRXR1* and *PcRXR4* quickly clustered with all the other crustaceans, and the clade of the crustacean group was more close to the clade of vertebrate group and separated it from other arthropods’ *RXRs* (Figure 3b).

### 2.3. Expression of PcEcR and PcRXR in Different Tissues

**Figure 4:**
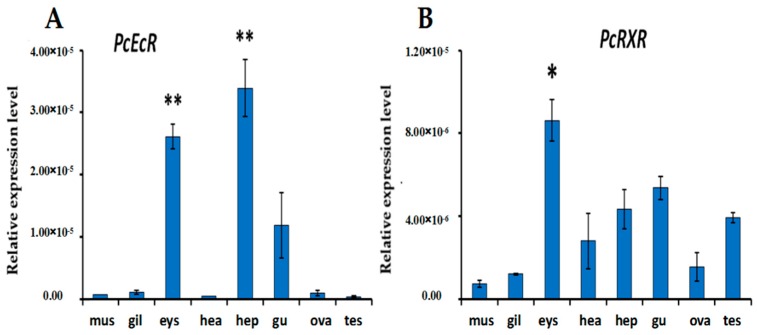
PCR analysis of relative expression levels of *PcEcR* (**A**) and *PcRXR* (**B**) in eight tissues of *P. clarkii*. mus: muscle; gil: gill; eys: eyestalk; hea: heart; hep: hepatopancreas; gu: gut; ova: ovary; tes: testis; Each data point represents the mean and standard deviation (*n* = 3 samples). The expression level in hepatopancreas was considerably higher than in other tissues (**: *p* < 0.01, *: *p* < 0.05; with Student’s *t*-test).

Both *PcEcR* and *PcRXR* were expressed in all eight tissues that were examined (Figure 4). It was observed that *PcEcR* was highly expressed in hepatopancreas and eyestalk, with the least expression in Testis. In the case of *PcRXR*, it was highly expressed in the eyestalk showing the lowest expression in muscle (Figure 4).

### 2.4. The Induction Expression of PcEcR and PcRXR after Eyestalk Ablation

**Figure 5:**
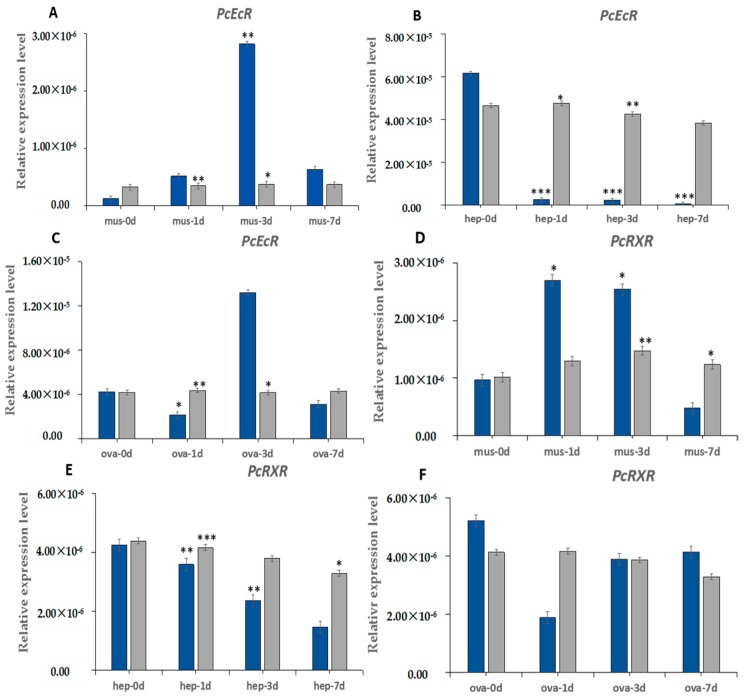
Expression level of *PcEcR* (**A**–**C**) and *PcRXR* (**D**–**F**) after eyestalk ablation in (**A**,**D**) muscle, in (**B**,**E**) hepatopancreas and in (**C**,**F**) ovary. *P. Clarkii:* mus: muscle; hep: hepatopancreas; ova: ovary; d: day. The blue represnts the expression by eyestalk ablation. The gray represents the expression without eyestalk ablation. Each data point represents the mean and standard deviation (*n* = 3 samples). Statistical analyses were performed with Student’s *t*-test (***: *p* < 0.001, **: *p* < 0.01, *: *p* < 0.05).

The expression of *PcEcR* and *PcRXR* were detected in three crayfish tissues at 0 days, 1 day, 3 days and 7 days after bilateral eyestalk ablation. As shown in Figure 5, the response of *PcEcR* and *PcRXR* to eyestalk ablation is different in different tissues. In muscle *PcEcR* and *PcRXR* were upregulated after ESA, *PcEcR* reached the highest level on day 3 after ESA and increased 33.5-fold relative to day 0, and *PcRXR* reached the highest level on day 1 after ESA and increased 2.7-fold relative to day 0. In hepatopancreas, *PcEcR* and *PcRXR* were d*EcR*eased continuously after ESA, the expression levels of *PcEcR* and *PcRXR* were only 0.7% and 1.7% in day 7 after ESA relative to day 0, respectively. In ovary, *PcEcR* were upregulated after ESA, reached the highest level on day 3 after ESA, and increased 3.0-fold relative to day 0, and the expression level of *PcRXR* changed insignificantly after ESA (*p* >0.05, Student’s *t*-test).

## 3. Discussion

### 3.1. Analysis of PcEcR and PcRXR

In the study, we have cloned two cDNAs encoding *PcEcR* and *PcRXR* from the red swamp crayfish. These play the role of an ecdysone receptor complex in *Procambarus clarkii*. They were found to be highly similar to known sequences of *EcR* and *RXR*/*USP* [21]. They also exhibit typical sequence domain structures of other *EcRs* and *RXRs* from insects and vertebrates [15]. The C domain, which is the DNA-binding domain, is the most conserved in both *EcRs* and *RXRs* [6]. DBD of *PcEcR* and *PcRXR* exhibits a high degree of identity with the other proteins. The E domain which is a ligand-binding domain, exhibit moderate conservation in both *EcRs* and *RXRs*. LBD of *PcEcR* and *PcRXR* shares a general degree of identity with the compared proteins. This supports the notion that *PcEcR* and *PcRXR* can perform the functions similar to the other proteins.

In the A/B domain, variant sequences exist in insects caused by alternative splicing. Their expressions are regulated by different promoters, resulting in the different expression pattern of isoforms in a tissue-specific manner. Similarly to insects, the variant regions in crustaceans occur in the A/B domain [15], the hinge domain and the ligand domain [11,13,18]. Variant sequences in these regions presumably affect dimerization, transcription activation, and ligand binding. However, we have found a short 15 bp insertion/deletion region, which encoded 5 amino acids occurring in the DNA binding domain. These variants are produced by alternative splicing, and their expression is regulated by distinct promoters. The variable regions may bear some functional significance(s) in *RXR* binding or action, as steric hindrance or rigidity of Pro may alter flexibility or conformation within the molecule and importance of *RXR* signal transactivation properties or ligand affinities. The insertion is present in *PcRXR1* and *PcRXR*3 while absent in *PcRXR*2 and *PcRXR4*. It suggests the DNA binding activity between *PcRXR1*, 3 and *PcRXR*2, 4 may be different. A similar pattern is found in other crustacean *RXRs*, although the size or its position of an insertion in DBD and/or LBD varies [20]. Further studies are required to know whether these multiple variants of *PcRXR* have different properties in ligand binding, DNA binding and heterodimerization.

In the phylogenetic tree of *EcRs*, the crustacean group is clustered in one clade and the insect group in another. This suggests that *PcEcR* is different from that of insects. In the phylogenetic tree of *RXRs*, *PcRXR1* and *PcRXR4* quickly clustered with all the other crustaceans, and the clade of the crustacean group was closer to the clade of vertebrate group and separated it from other arthropods’ *RXRs*. The vertebrate *RXR* binds retinoic acid preferentially and forms a homodimer. In contrast, the insect *RXR* has been identified as an orphan receptor, although a putative ligand, juvenile hormone, bound to *RXR* at higher concentrations than those causing physiological effects. The retinoic acid is the ligand of *PcRXR* in which LBD is highly conserved in that of crustacean *RXR* [13]. Thus, the crustacean *RXR* is closer to vertebrate *RXR* than to insect *RXR*.

### 3.2. Expression in Different Tissues and Synergistic Expression of PcEcR and PcRXR in Different Tissues

All internal tissues in crustaceans can be considered to be the target tissues of hemolymphatic ecdysteroids. All these tissues exhibit the co-presence of *EcR*/*RXR* expression, supporting the notion that they act as a heterodimer. However, the levels of their expression vary in different tissues with different levels of *PcEcR* and *PcRXR* expression. Both *PcEcR* and *PcRXR* were expressed in all eight tissues examined. It was observed that *PcEcR* was highly expressed in the hepatopancreas with the least expression in Testis. *PcEcR* was highly expressed in the hepatopancreas, which is consistent with *EcR* in *Macrobrachium nipponense* and *EcR* in *Eriocheir sinense*. The hepatopancreas is the major organ related to metabolism in animals; high expression levels of *PcEcR* in the hepatopancreas indicate that *EcR* is necessary for development in crayfish [6]. In the case of *PcRXR*, it was highly expressed in the eyestalk with the least expression in muscle. *PcRXR* was highly expressed constantly compared with *PcEcR* in testis and ovary indicating the possibility that *PcRXR* is other than ecdysteroids was required for development and maturation of reproductive tissues. For example, the expression of *CpRXR* and *MjRXR* gradually increased during ovarian maturation, which supports the importance of *RXR* in reproduction [13]. Reproduction in crustaceans is closely related to molting, and the underlying mechanism of reproductive processes is not yet well-understood.

### 3.3. Induction of PcEcR and PcRXR after ESA in Different Tissues

This is because the X-organ/sinus complex is located in the eyestalk [12,25,26,27,28,29]. Several important neuropeptides including MIH (molt-inhibiting hormone), gonad/vitellogenesis-inhibiting hormone, crustacean hyperglycemic hormone, and mandibular organ-inhibiting hormone are s*EcR*eted by the X-organ/sinus complex in crustaceans [30,31]. These neuropeptide hormones regulate multiple physiological processes, such as metabolism, reproduction, and osmoregulation [32,33,34]. Also, it s*EcR*etes gonad-inhibiting hormone to regulate gonadal dysgenesis. Eyestalk ablation breaks the X organ-sinus gland complex functions or weakens it. Therefore, eyestalk ablation promotes molting and growth.

The response of *PcEcR* and *PcRXR* to eyestalk ablation is different in different tissues. In the hepatopancreas, *PcEcR* and *PcRXR* d*EcR*ease continuously after ESA. Both of them were upregulated in muscle and ovaries in general. The induction of gonad maturation and the molting results are affected by *EcR* and *RXR* after ESA. The process described for the *EcR* and *RXR* gene is indeed involved in molting. The effect of ecdysteroids is mediated by a receptor complex composed of ecdysone receptor (*EcR*) and retinoid X receptor (*RXR*) homolog in crustaceans. Overall, the *PcEcR*/*PcRXR* complex functions as a mediator of ecdysteroid signals. The hepatopancreas plays the role of a positive regulator in molting and reproduction. However, *PcEcR* and *PcRXR* were not upregulated continuously after ESA in muscle and ovary. The expression patterns of *EcR* and *RXR* did not coincide with the process of ecdysteroid titer and were different depending on different times. These imply that the expression of these genes was not controlled by ecdysteroid only. The expressions were also affected by the other factors. Also, a similar result was observed in Eriocheir sinensis [24]. The variable effect in different tissues after ESA indicates different tissues may have a notable difference in sensitivity to the concentration and a specific type of Ecds and may coordinate their inherent specific functions during molting and gonad maturation.

## 4. Material and Methods

### 4.1. Animal Collection, Preparation of Total RNA, and cDNA Synthesis

Crayfish *P. clarkii* that were about 10–20 grams weight were collected from a crayfish farm in Xuyi, Jiangsu Province, China. They were cultured in water tanks with adequate aeration at 20 °C in a natural photoperiod and fed with a commercial crayfish diet once a day. The methods of eyestalk ablation can be subdivided into two: unilateral resection and bilateral resection. The effects of bilateral resection are fast and significant but the mortality rate is high because the endocrine is not in control. Molting in the unilateral resection group is slower in comparison to that of the bilateral group [35]. The practice show that bilateral resection tend to have higher survival rates. In this here study, the experiments were conducted with respect to the bilateral resection due to its effects. In order to establish the expression levels, samples were collected from different tissues from 3 crayfish (1male and 2 female). For the eyestalk ablation experiment, crayfish (female) in the intermolt stage were chosen for the ablation of bilateral eyestalk using sterile surgical scissors. The same samples from different tissues were collected from crayfish at 0, 1, 3 and 7 days after eyestalk ablation. Tissue samples were frozen immediately in liquid nitrogen and then stored at −80 °C.

Total RNA from various tissues was isolated using the TRIzol^®^ Reagent (Invitrogen, Waltham, MA, USA) according to the manufacturer’s protocol. RNA integrity was evaluated by 1.5% agarose gel electrophoresis. The concentrations were measured and the purity of the RNA was determined by use of a NanoDrop ND-1000 spectrophotometer (NanoDrop Technologies, Wilmington, DE, USA). cDNA was synthesized according to the manufacturer’s protocol using the SuperScript II RNase H reverse transcriptase first strand synthesis system (Invitrogen, Waltham, MA, USA).

### 4.2. Cloning and Sequencing of Full-Length PcEcR and PcRXR cDNA

Two partial cDNA sequences highly similar to published *EcRs* and *RXRs* (cDNA in Genbank) from our deep sequencing data were identified, respectively. Based on these two partial cDNA sequences, gene-specific 3′ and 5′ primers were designed for RACE PCR (rapid amplification of cDNA ends) (Table 1). 3′ and 5′ RACE cDNA were prepared from total RNA of *P. clarkii* (hepatopancreas), using a 3′-Full RACE Core Set Ver.2.0 Kit and 5′-Full RACE kit (Takara, Dalian, China) according to the manufacturer’s instructions, respectively. After performing two rounds of PCR to obtain 3′ and 5′ end fragments of *PcEcR* and *PcRXR*, the final PCR products were cloned into the pEASY-T1 vector (Transgen, Beijing, China). The recombinant plasmids were used to transform *E. coli* (*Escherichia coli*) TOP 10 competent cells, isolated, and sequenced.

### 4.3. Sequence Alignments and Phylogenetic Analysis

The deduced amino acid sequences of *Procambrus clarkii EcR* were aligned with the seven known *EcRs* of other species, derived from the NCBI GenBank database: *AsEcR* from *Ascaris suum EcR* (ADY42041.1), *BmEcR* from *Bombyx mori* (BAA07890.1), *CcEcR* from *Crangon crangon* (Accession Number ACO44665.1), *CgEcR* from *Crassostrea gigas EcR* (EKC19773.1), *DamEcR* from *Daphnia magna* (BAF49029.1), *DmEcR* from *Drosophila melanogaster* (AAF57278.3), and *EsEcR-L* from *Eriocheir sinensis* (KF469222). A neighbor-joining tree was constructed from multiple sequence alignments with 16 other *EcR* protein sequences derived from the GenBank database using the molecular evolutionary genetics analysis (MEGA) software, version 3.1 (www.mega.co.nz). Bootstrap analysis of 1000 replicates was carried out to determine the confidence of tree branch positions. The names and the accession numbers of the *EcR* proteins used are as follows: *Crangon crangon EcR* (Accession Number ACO44665.1), *Daphnia magna EcR* (BAF49029.1), *Eriocheir sinensis EcR-L* (KF469222), *Macrobrachium nipponense EcR* (KC631613), *Marsupenaeus japonicus EcR* (Accession Number: BAF75375.1), *Portunus trituberculatus EcR* (AFH35032.1), *Uca pugilator EcR* (AAC33432.2), *Aedes aegypti EcR* (XP_001660279.1), *Apis mellifera EcR* isoform A (NP_001091685.2), *Bombyx mori EcR* (BAA07890.1), *Drosophila melanogaster EcR* (AAF57278.3), *Tenebrio molitor EcR* (CAA72296.1), *Tribolium castaneum EcR* isoform A (NP_001107650.1), *Ascaris suum EcR* (ADY42041.1), *Crassostrea gigas EcR* (EKC19773.1), and *Trichinella spiralis EcR* (XP_003376657.1).

The deduced amino acid sequences of *Procambrus clarkii RXR* were aligned with the six known *RXRs* of other species, derived from the NCBI GenBank database: *MjRXR* from *Marsupenaeus japonicus* (Accession Number: BAF75376), *DamRXR* from *Daphnia magna* (ABF74729), *DmUSP* from *Drosophila melanogaster* (NP_476781), *BmUSP* from *Bombyx mori* (NP_001037470), Dr*RXR* from *Danio rerio RXR*-γ-A (NP_571292) and Hs*RXR*A from *Homo sapiens* (AAH63827). *PcRXR1*, *PcRXR4* and 18 *RXR* proteins from other species were involved in neighbor-joining tree construction, their names and the accession numbers are as follows: *Celuca pugilator RXR* homolog (AAC32789), *Crangon crangon RXR* isoform 1 (Accession Number: ACO44668), *Fenneropenaeus chinensis RXR* (1130559), *Gecarcinus lateralis RXR*a (AAZ20368), *Marsupenaeus japonicus RXR* (BAF75376), *Aedes aegypti USP* isoform-A (AAG24886), *Apis mellifera USP* isoform-A (AAF73057), *Blattella germanica RXR* (CAH69897), *Bombyx mori USP* (NP_001037470), *Danio rerio RXR*-γ-A (NP_571292), *Drosophila melanogaster USP* (NP_476781), *Homo sapiens RXR*A (AAH63827), *Macrobrachium nipponense RXR*-L (KC460323), *Marsupenaeus japonicus RXR* (Accession Number: BAF75376), *Mus musculus RXR* α 2 (AAB36777), *Tenebrio molitor USP* protein (CAB75361), *Tribolium castaneum USP* (CAL25729), and *Xenopus laevis RXR*b-a (AAI08461).

### 4.4. Quantitation of PcEcR and PcRXR Transcripts by Real-Time PCR

The quantitative real-time PCR assay was performed using the ABI 7500 system (Applied Biosystems, New York, NY, USA) to detect the expression levels of *PcEcR* and *PcRXR*. The expression of the 18S RNA gene of *Procambrus clarkii* (accession number: EU920952.1) was selected as the reference gene to be an internal and experiment control, using the primer pair *Pc18S*-F and *Pc18S*-R. The primers Rt-*PcEcR*-F and Rt-*PcEcR*-R were designed to detect the expression of *PcEcR*, and primers Rt-*PcRXR*-F and Rt-*PcRXR*-R were designed to detect the expression of *PcRXR*. The real-time PCR program was run at a temperature 95 °C for 3 min, 40 cycles of 95 °C for 10 s, 60 °C for 20 s and 72 °C for 34 s. PCR reactions were performed in triplicate for each sample, and the expression levels were normalized to that of the 18S RNA gene. All the primers used for quantitative real-time PCR are listed in Table 1.

## Figures and Tables

**Table 1:** Nucleotide sequences of primers for *PcEcR* and *PcRXR* cloning and expression analysis.

Primer	Sequence (5′ to 3′)	Primer Description
*PcEcR*-3a-Outer	CTCACAGAATTGCGAACCCTT	3′ RACE Primer for first round
*PcEcR*-3a-Inner	CACCCAGGACCCACTTTCAG	3′ RACE Primer for second round
*PcEcR*-5a-Outer	GAGATGTTACTGCTTCCCAC	5′ RACE Primer for first round
*PcEcR*-5a-Inner	ATCCTTTGGGTTTACAATCA	5′ RACE Primer for second round
*PcRXR*-3a-Outer	CAATACTGGCATCGGTTCCT	3′ RACE Primer for first round
*PcRXR*-3a-Inner	CCGCCATTGGTGGTGGAGAA	3′ RACE Primer for second round
*PcRXR*-5a-Outer	AAAACAAGGAAGTAGTTGGC	5′ RACE Primer for first round
*PcRXR*-5a-Inner	TAAAACTCAAGGAAACTGATG	5′ RACE Primer for second round
Rt-*PcEcR*-F	CCTGTGAGGGATGCAAAGGT	FWD primer for *EsEcR* expression
Rt-*PcEcR*-R	GCATTGAGACTCGGGAACCA	RVS primer for *EsEcR* expression
Rt-*PcRXR*-F	CCTTCACCATTGGGTCGAGT	FWD primer for *EsEcR* expression
Rt-*PcRXR*-R	AGCTGTAGACGCCATAGTGC	RVS primer for *EsEcR* expression
*Pc18S*-F	ATCACGTCTCTGACCGCAAG	FWD primer for 18S expression
*Pc18S*-R	GACACTTGAAAGATGCGGCG	RVS primer for 18S expression

## References

[1] Yue G.H., Wang G.L., Zhu B.Q., Wang C.M., Zhu Z.Y., Lo L.C. (2008). Discovery of four natural clones in a crayfish species *Procambarus clarkii*. Int. J. Biol. Sci..

[2] Wang W., Gu W., Ding Z., Ren Y., Chen J., Hou Y. (2005). A novel Spiroplasma pathogen causing systemic infection in the crayfish *Procambarus clarkii* (Crustacea: Decapod), in China. FEMS Microbiol. Lett..

[3] Nakagawa Y., Henrich V.C. (2009). Arthropod nuclear receptors and their role in molting. FEBS J..

[4] Yao T.P., Forman B.M., Jiang Z., Cherbas L., Chen J.D., McKeown M., Cherbas P., Evans R.M. (1993). Functional ecdysone receptor is the product of *EcR* and ultraspiracle genes. Nature.

[5] Kim H.W., Lee S.G., Mykles D.L. (2005). Ecdysteroid-responsive genes, *RXR* and E75, in the tropical land crab, Gecarcinus lateralis: Differential tissue expression of multiple *RXR* isoforms generated at three alternative splicing sites in the hinge and ligand-binding domains. Mol. Cell. Endocrinol..

[6] Shechter A., Tom M., Yudkovski Y., Weil S., Chang S.A., Chang E.S., Chalifa-Caspi V., Berman A., Sagi A. (2007). Search for hepatopancreatic ecdysteroid-responsive genes during the crayfish molt cycle: From a single gene to multigenicity. J. Exp. Biol..

[7] King-Jones K., Charles J.P., Lam G., Thummel C.S. (2005). The ecdysone-induced DHR4 orphan nuclear receptor coordinates growth and maturation in Drosophila. Cell.

[8] Nikolenko J.V., Krasnov A.N. (2007). Nuclear receptors: structure and mechanisms of action. Genetika.

[9] Renaud J.P., Moras D. (2000). Structural studies on nuclear receptors. Cell. Mol. Life Sci..

[10] Thomson S.A., Baldwin W.S., Wang Y.H., Kwon G., Leblanc G.A. (2009). Annotation, phylogenetics, and expression of the nuclear receptors in Daphnia pulex. BMC Genom..

[11] Chung A.C., Durica D.S., Clifton S.W., Roe B.A., Hopkins P.M. (1998). Cloning of crustacean ecdysteroid receptor and retinoid-X receptor gene homologs and elevation of retinoid-X receptor mrna by retinoic acid. Mol. Cell. Endocrinol..

[12] Kim H.W., Chang E.S., Mykles D.L. (2005). Three calpains and ecdysone receptor in the land crab gecarcinus lateralis: Sequences, expression and effects of elevated ecdysteroid induced by eyestalk ablation. J. Exp. Biol..

[13] Asazuma H., Nagata S., Kono M., Nagasawa H. (2007). Molecular cloning and expression analysis of ecdysone receptor and retinoid X receptor from the kuruma prawn, marsupenaeus japonicus. Comp. Biochem. Physiol. B Biochem. Mol. Biol..

[14] Kato Y., Kobayashi K., Oda S., Tatarazako N., Watanabe H., Iguchi T. (2007). Cloning and characterization of the ecdysone receptor and ultraspiracle protein from the water flea daphnia magna. J. Endocrinol..

[15] Hwang D.S., Lee J.S., Lee K.W., Rhee J.S., Han J., Lee J., Park G.S., Lee Y.M. (2010). Cloning and expression of ecdysone receptor (*EcR*) from the intertidal copepod, *Tigriopus japonicus*. Comp. Biochem. Physiol. C Toxicol. Pharmacol..

[16] Verhaegen Y., Parmentier K., Swevers L., Renders E., Rouge P., de Coen W., Cooreman K., Smagghe G. (2011). The heterodimeric ecdysteroid receptor complex in the brown shrimp *Crangon crangon*: *EcR* and *RXR* isoform characteristics and sensitivity towards the marine pollutant tributyltin. Gen. Comp. Endocrinol..

[17] Yokota H., Eguchi S., Nakai M. (2011). Development of an *in vitro* binding assay for ecdysone receptor of mysid shrimp (*Americamysis bahia*). Aquat. Toxicol..

[18] Tarrant A.M., Behrendt L., Stegeman J.J., Verslycke T. (2011). Ecdysteroid receptor from the american lobster *Homarus americanus*: *EcR*/*RXR* isoform cloning and ligand-binding properties. Gen. Comp. Endocrinol..

[19] Gaertner K., Chandler G.T., Quattro J., Ferguson P.L., Sabo-Attwood T. (2012). Identification and expression of the ecdysone receptor in the harpacticoid copepod, amphiascus tenuiremis, in response to fipronil. Ecotoxicol. Environ. Saf..

[20] Techa S., Chung J.S. (2013). Ecdysone and retinoid-X receptors of the blue crab, *Callinectes sapidus*: Cloning and their expression patterns in eyestalks and Y-organs during the molt cycle. Gene.

[21] De Wilde R., Swevers L., Soin T., Christiaens O., Rouge P., Cooreman K., Janssen C.R., Smagghe G. (2013). Cloning and functional analysis of the ecdysteroid receptor complex in the opossum shrimp neomysis integer (leach, 1814). Aquat. Toxicol..

[22] Shen H., Zhou X., Bai A., Ren X., Zhang Y. (2013). Ecdysone receptor gene from the freshwater prawn macrobrachium nipponense: Identification of different splice variants and sexually dimorphic expression, fluctuation of expression in the molt cycle and effect of eyestalk ablation. Gen. Comp. Endocrinol..

[23] Shen H., Zhou X., Bai A., Ren X. (2013). cDNA cloning of retinoid-X receptor gene from macrobrachium nipponense and different responses of two splice variants during the molting cycle. CRUS.

[24] Shen H., Hu Y., Zhou X. (2015). Cloning of ecdysone receptor gene from the Chinese Mitten Crab, *Eriocheir sinensis* and sexually dimorphic expression of two splice variants. J. World Aquac. Soc..

[25] Uawisetwathana U., Leelatanawit R., Klanchui A., Prommoon J., Klinbunga S., Karoonuthaisiri N. (2011). Insights into eyestalk ablation mechanism to induce ovarian maturation in the black tiger shrimp. PLoS ONE.

[26] McDonald A.A., Chang E.S., Mykles D.L. (2011). Cloning of a nitric oxide synthase from green shore crab, carcinus maenas: A comparative study of the effects of eyestalk ablation on expression in the molting glands (Y-organs) of C. maenas, and blackback land crab, Gecarcinus lateralis. Comp. Biochem. Physiol. A Mol. Integr. Physiol..

[27] Sroyraya M., Chotwiwatthanakun C., Stewart M.J., Soonklang N., Kornthong N., Phoungpetchara I., Hanna P.J., Sobhon P. (2010). Bilateral eyestalk ablation of the blue swimmer crab, portunus pelagicus, produces hypertrophy of the androgenic gland and an increase of cells producing insulin-like androgenic gland hormone. Tissue Cell.

[28] Okumura T. (2007). Effects of bilateral and unilateral eyestalk ablation on vitellogenin synthesis in immature female kuruma prawns, marsupenaeus japonicus. Zool. Sci..

[29] Venkitraman P.R., Jayalakshmy K.V., Balasubramanian T., Nair M., Nair K.K. (2004). Effects of eyestalk ablation on moulting and growth of penaeid prawn metapenaeus dobsoni (de Man). Indian J. Exp. Biol..

[30] Zhang Y., Sun Y., Liu Y., Geng X., Wang X., Wang Y., Sun J., Yang W. (2011). Molt-inhibiting hormone from Chinese mitten crab (Eriocheir sinensis): Cloning, tissue expression and effects of recombinant peptide on ecdysteroid s*EcR*etion of YOs. Gen. Comp. Endocrinol..

[31] Webster S.G., Keller R., Dircksen H. (2012). The CHH-superfamily of multifunctional peptide hormones controlling crustacean metabolism, osmoregulation, moulting, and reproduction. Gen. Comp. Endocrinol..

[32] Sedlmeier D. (1982). The mode of action of the crustacean neuros*EcR*etory hyperglycemic hormone (CHH): II. Involvement of glycogen synthase. Gen. Comp. Endocrinol..

[33] Soyez D., Le Caer J.P., Noel P.Y., Rossier J. (1991). Primary structure of two isoforms of the vitellogenesis inhibiting hormone from the lobster Homarus americanus. Neuropeptides.

[34] Spanings-Pierrot C., Soyez D., van Herp F., Gompel M., Skaret G., Grousset E., Charmantier G. (2000). Involvement of crustacean hyperglycemic hormone in the control of gill ion transport in the crab Pachygrapsus marmoratus. Gen. Comp. Endocrinol..

[35] Yin H. (2007). Effects of eyestalk ablation on the molting, growth and gonad development of *Procambarus clarkia*. J. Anhui Agric. Sci..

